# Manganese Ions Individually Alter the Reverse Transcription Signature of Modified Ribonucleosides

**DOI:** 10.3390/genes11080950

**Published:** 2020-08-18

**Authors:** Marco Kristen, Johanna Plehn, Virginie Marchand, Kristina Friedland, Yuri Motorin, Mark Helm, Stephan Werner

**Affiliations:** 1 Institute of Pharmaceutical and Biomedical Sciences, Johannes Gutenberg, University Mainz, Staudingerweg 5, 55128 Mainz, Germany; makriste@uni-mainz.de (M.K.); jplehn@uni-mainz.de (J.P.); kfriedla@uni-mainz.de (K.F.); mhelm@uni-mainz.de (M.H.); 2 Epitranscriptomics and RNA Sequencing (EpiRNA-Seq) Core Facility, UMS2008 IBSLor CNRS, Université de Lorraine-INSERM, Biopôle, 9 Avenue de la Forêt de Haye, 54505 Vandœuvre-lès-Nancy, France; virginie.marchand@univ-lorraine.fr (V.M.); yuri.motorin@univ-lorraine.fr (Y.M.); 3 IMoPA, UMR7365 CNRS, Université de Lorraine, Biopôle, 9 Avenue de la Forêt de Haye, 54505 Vandœuvre-lès-Nancy, France

**Keywords:** reverse transcription, RT signature, RNA modifications, m^1^A, manganese chloride

## Abstract

Reverse transcription of RNA templates containing modified ribonucleosides transfers modification-related information as misincorporations, arrest or nucleotide skipping events to the newly synthesized cDNA strand. The frequency and proportion of these events, merged from all sequenced cDNAs, yield a so-called RT signature, characteristic for the respective RNA modification and reverse transcriptase (RT). While known for DNA polymerases in so-called error-prone PCR, testing of four different RTs by replacing Mg^2+^ with Mn^2+^ in reaction buffer revealed the immense influence of manganese chloride on derived RT signatures, with arrest rates on m^1^A positions dropping from 82% down to 24%. Additionally, we observed a vast increase in nucleotide skipping events, with single positions rising from 4% to 49%, thus implying an enhanced read-through capability as an effect of Mn^2+^ on the reverse transcriptase, by promoting nucleotide skipping over synthesis abortion. While modifications such as m^1^A, m^2^_2_G, m^1^G and m^3^C showed a clear influence of manganese ions on their RT signature, this effect was individual to the polymerase used. In summary, the results imply a supporting effect of Mn^2+^ on reverse transcription, thus overcoming blockades in the Watson-Crick face of modified ribonucleosides and improving both read-through rate and signal intensity in RT signature analysis.

## 1. Introduction

Research on RNA modifications has experienced an impressive renaissance in the last few years, driven by a rapid growth in the field of epitranscriptomic research [1] and based on ever improving in vitro and in silico methods. Some modifications have been associated with various human diseases, including neurological disorders and multiple types of cancer [2], thus showing the necessity of fast and reliable detection methods. Typically, laboratory work based on different chemical agents and modification-specific treatment is combined with high-performance bioinformatical systems, able to process huge amounts of sequencing data in automated pipelines, resulting in what is known as modification calling [3].

Ever since the discovery of pseudouridine as a posttranscriptional modification in the 1950s [4], over 160 different modified nucleosides have been identified in RNA [5]. Dependent on organism and RNA species, modifications vary in their frequency, being crucial for functionality of highly abundant and extensively modified tRNA [6] or major players in mRNA-associated regulation of gene expression [7]. Additionally, a wide range of alterations, from single methylations on adenosine as in m^1^A or m^6^A, up to complex modifications comprising multiple steps of formation and rare biological structures such as a thioacetal [8] has been identified. From this variety, several modifications are considered key factors in cancer research, hence emphasizing the importance of m^1^A, m^6^A, m^5^C, inosine and pseudouridine [9]. To analyze the prevalence of these modifications, several methods have emerged, dependent on the chemical properties of the respective nucleoside. Therefore, one approach uses the altered transcriptional behavior of modifications such as m^1^A, an adenosine bearing a methyl group in the Watson-Crick face, thus impeding conventional base pairing. This promotes incorporation of non-complementary deoxynucleoside triphosphates (dNTPs) in reverse transcription [10], or even arrest of primer elongation. According to the necessity of converting RNA into DNA for current RNA-seq methods, apart from direct RNA sequencing by nanopore technology [11,12], a combination of suitable library preparation protocols that allow capture of abortive cDNA fragments [13,14] and use of advanced bioinformatical pipelines [15], is needed for efficient analysis of modification prevalence in RNA species [16]. However, most modified nucleosides do not impede reverse transcription to a detectable extent, implying that information about modification type and sequence context may get lost due to the limited vocabulary of only four canonical deoxynucleotides in cDNA synthesis. To conserve this information throughout reverse transcription, modification specific enzymatic [17,18] or chemical treatments [3,19] are applied. Methods such as PSI-seq [19] or AlkAniline-seq [20] are used. The latter combines alkaline treatment of template RNA with subsequent aniline cleavage and advanced bioinformatical processing, thereby analyzing accumulation of abortive cDNA fragments that end at the former modification sites, allowing detection of m^7^G and m^3^C positions. Moreover, other methods, for instance bisulfite sequencing [21], use the protective nature of certain modifications against chemical agents, thus preventing base conversion of this site. Accordingly, the modification site differs in cDNA by bearing the respective canonical nucleoside, while all non-modified positions have been altered. In conclusion, a wide range of methods for the detection of RNA modifications are based on misincorporation of dNTPs into cDNA and capture of abortive cDNA fragments, thereby drawing a specific pattern for each modification, so-called RT signature. Combined with advanced bioinformatical systems of deep learning [22] and automated workflows [15], fast and reliable analysis of RNA modifications is possible for a wide range of modifications [23].

In addition to primer extension arrest and dNTP misincorporation, an event occurring in reverse transcription of modified nucleosides that has only recently been described [16] focuses on the ability of polymerases to skip certain nucleotides, visible as deletion in sequencing data. While the occurrence in a modification context is rather uncharacterized, nucleotide skipping is well-known in the field of RNA splicing research [23,24] and probably based on a slipped-strand mispairing mechanism [25]. This feature, termed jump, is visible in cDNA as deletion of one or two nucleotides at or shortly after a modification-associated site and proved suitable as an influential parameter of RT signature in modification calling [16]. However, the RT signature is dependent on multiple factors, comprising the kind of bases preceding the modification site, type of modification, the polymerase used and reaction conditions [16,21]. Altered transcription conditions are commonly used for error-prone PCR, a method by which random mutations are introduced to the newly synthesized DNA strand by adding MnCl_2_ to the reaction buffer, thus lowering sequence-fidelity of the polymerase [26]. Comparable effects have been observed in reverse transcription under Mn^2+^ addition, resulting in more erroneous cDNA, bearing increased levels of misincorporations and deletions [27]. Additionally, Mn^2+^ is known to decrease activity and processivity of reverse transcriptases at elevated concentrations when compared to Mg^2+^ and a wide range of other divalent cations [28,29]. These effects are individual to the respective polymerase and highly dependent on the manganese concentration, with some RTs displaying a magnesium-like transcription performance at low Mn^2+^ concentrations, but increasingly impaired fidelity and processivity at elevated levels [30,31].

Here, we present an RT signature analysis of eukaryotic tRNA, combining modified transcription conditions, multiple polymerases and an extended RT feature set. The analysis revealed a profound influence of manganese on RT signature, and furthermore highly individual signature-alteration for different modified nucleosides. Demonstrated on m^1^A, levels of misincorporation and jump increased distinctly, whereas significantly less abortive cDNA could be observed, while dependent on manganese concentration and polymerase type. These effects could be used to strengthen RT signature-derived signals not only directly via increased levels of misincorporation and jump, but also through reduced arrest rates in protocols not able to capture abortive cDNA fragments.

## 2. Materials and Methods

### 2.1. Sample Treatment and Library Preparation

**Figure 1:**
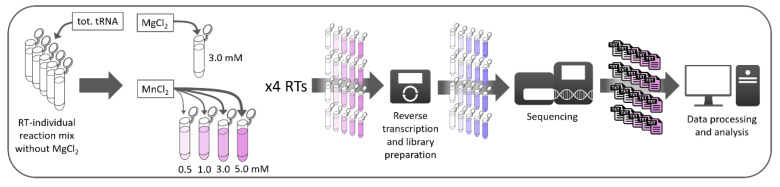
Overview of the sample preparation, comprising individual reaction mixtures for every transcriptase and addition of either MgCl_2_ (reference) or four concentrations of MnCl_2_, thereby resulting in a total of 20 distinct reaction conditions.

Library preparation was derived from a previously published protocol [22], allowing capture of abortive cDNA fragments that occur in reverse transcription, therefore crucial for the investigation of RNA modifications that cause primer extension arrest. In brief, sample preparation comprised dephosphorylation of total tRNA of *Saccharomyces cerevisiae* from Roche (Sigma-Aldrich, Darmstadt, Germany) with FastAP (thermosensitive Alkaline Phosphatase; Thermo Fisher Scientific, Dreieich, Germany); afterwards, ligation of a pre-adenylated 3’-adapter using T4 RNA Ligase (Thermo Fisher Scientific) and T4 RNA Ligase 2 truncated (New England Biolabs, Frankfurt am Main, Germany) and subsequent steps of purification with 5’-Deadenylase (New England Biolabs) and Lambda exonuclease (Thermo Fisher Scientific) were carried out to remove non-ligated pre-adenylated primer. Following ethanol precipitation and resuspension of the RNA pellet, a modified reverse transcription was performed. Therefore, the supplier’s RT buffer was replaced with custom made buffers for each transcriptase (SuperScript III and SuperScript IV: Thermo Fischer Scientific; ProtoScript II: New England Biolabs; EpiScript; Lucigen, Middleton WI, USA). The standard buffer composition was used as reference. Additionally, four versions replacing MgCl_2_ (3 mM) with differing concentrations of MnCl_2_ (0.5 mM, 1.0 mM, 3.0 mM, 5.0 mM) were prepared for each transcriptase, resulting in a total of 20 distinct reaction conditions. Reverse transcription was carried out on equal amounts of the previously treated total tRNA, using the four reverse transcriptases in their respective buffers. After reverse transcription, remaining RT primer was digested using Lambda exonuclease and Exonuclease I (Thermo Fisher Scientific), followed by dephosphorylation of remnant dNTPs with FastAP thermosensitive Alkaline Phosphatase (Thermo Fisher Scientific). Next, samples were treated with NaOH to degrade remaining RNA, afterwards neutralized with acetic acid and again ethanol precipitated. Following 3’-tailing with TdT (terminal deoxynucleotidyl transferase; Thermo Fisher Scientific), DNA adapter ligation with T4 DNA Ligase (Thermo Fisher Scientific), a size selection via PAGE gel electrophoresis in the range of 100 to 150 nucleotides and a further step of ethanol precipitation, Illumina-required sequences were introduced via PCR, using P5 and P7 PCR primers and Taq-Polymerase (Rapidozym, Berlin, Germany). Once again precipitated, PCR products were purified on a 10% denaturing polyacrylamide gel by size selection in the range of 200 to 300 nucleotides. Finally, prepared libraries were quality controlled for adapter dimer formation on an Agilent 4200 TapeStation and afterwards fluorometrically quantified using the Qubit dsDNA HS assay kit (Thermo Fischer Scientific). Controlled samples were then analyzed on an Illumina MiSeq platform in 2 × 75 bp paired-end mode, demultiplexed and the resulting FastQ files used for further analysis. A graphical overview of sample preparation is shown in Figure 1.

### 2.2. Processing of Sequencing Output

Received sequencing output was processed on a local distribution of Galaxy [32] with an automated graphical workflow system deriving from Schmidt et al. [15]. This system allowed for combination of different bioinformatical tools and modules, seamlessly connected among themselves, thus enabling fast and precise processing of sequencing data. To meet the differing requirements in data treatment for reverse and forward reads of the paired-end sequencing, both reads were processed separately throughout the workflow. First, all FastQ files were quality-controlled using FastQC [33] and afterwards trimmed with Cutadapt [34], thus removing any remaining Illumina-related adapter sequences. Next, the trimmed reads were aligned to the reference sequences (total tRNA sequences for *S. cerevisiae* from Modomics [5] and tRNAdb [35]) using Bowtie 2 [36], thereby taking the high modification prevalence in tRNAs and the proposed erroneous influence of manganese chloride into account by allowing one mismatch within a 22 nucleotide sequence. Next, mapped sequences of the forward and reverse read were merged to a single file and subjected to final treatment. Here, we used custom python scripts, implemented in the workflow, in order to remove remnant bases from the 3’-tailing and creating a so-called profile file. The latter comprised single-base resolution tables, covering all positions in tRNAs from *S. cerevisiae* for each sample and bearing several descriptive features:

Coverage: Number of reads that were mapped to the reference position Arrest rate: Relative reduction in coverage compared to the neighboring (N+1) position. Mismatch rate: Relative number of mapped bases not matching the reference nucleotide at the given position. Jump rate: Relative amount of deletions occurring at or directly after the respective position (N-1). A distinction is made between deletions at the current position in reference (single jump direct), deletions at the neighboring 5’ (N-1) position (single jump delayed), and deletions on both the current and neighboring position (double jump).

Additionally, reference name (ref_seg), reference position (pos), reference base (ref_base) and coverage at the respective position were shown in the profile. Complementary, detailed information on the number of aligned bases by type (A, C, G, T) and unknown read bases (N), differentiated for forward (capital letters—A, C, G, T, N) and reverse reads (small letters—a, c, g, t, n), as well as the 3’-preceding base (prebase) were included. An exemplary profile file is shown in Table 1, comprising 10 m^1^A positions and the full set of descriptive features. A graphical overview of RT signature extraction (Figure S1) and manganese-derived alteration of modification profiles (Figure S2) can be found in the supplement.

Data analysis comprised annotation of modified sites, as well as filtering and screening of profile files. First, modified positions were annotated in accordance with Modomics [5] and the tRNAdb [35], followed by a filtration step, thereby discarding all positions with a coverage below 20 in order to ensure data quality. Next, the resulting profile files were screened for every modification type and non-modified positions, taking all previously described features (Table 1) into account. Finally, comprehensive visualizations of statistical parameters were created for every modification type, using custom R scripts.

## 3. Results

To investigate how manganese ions influence cDNA synthesis in reverse transcription, we processed total tRNA from *S. cerevisiae* with different RTs, using various concentrations of MnCl_2_. Thereupon, obtained RT products were inspected for Mn^2+^ derived alteration by deep sequencing, focusing on m^1^A, m^2^_2_G and other modified nucleosides. These naturally occurring modifications bear methyl groups that impact the ability to participate in Watson-Crick base pairing, thus frequently leading to erroneous RT products, suitable for RNA modification analysis.

First, reverse transcription was carried out in custom RT buffers at four different concentrations of MnCl_2_ (0.5 mM, 1.0 mM, 3.0 mM and 5.0 mM) in combination with four different polymerases (SuperScript III, SuperScript IV, ProtoScript II, EpiScript). A 0 mM Mn^2+^ sample served as reference of standard composition, containing 3 mM MgCl_2_, while other samples contained MnCl_2_ instead. Accordingly, 0 mM Mn^2+^ (=3 mM Mg^2+^) and 3 mM Mn^2+^ conditions were used for direct comparison, with other concentrations testing for reduced (0.5, 1.0 mM) and increased (5.0 mM) manganese influence. Next, an Illumina compatible library was prepared from obtained cDNA, comprising subsequent steps of phosphatase treatment, tailing and Illumina adapter ligation, using a protocol previously described by Hauenschild et al. [22]. The sequencing-ready library was then analyzed on an Illumina MiSeq platform in paired-end mode and the obtained data processed with a local Galaxy distribution, as presented by Schmidt et al. [15]. Data treatment included quality control, removal of Illumina-derived sequences and alignment to the tRNA reference.

To simplify downstream analysis, aligned reads were further processed with custom scripts, setting up tables with single base resolution in so-called profile files and discarding all positions with a coverage below 20, thereby ensuring data quality. These files contained detailed information on all positions in *S. cerevisiae* tRNA, comprising the number of sequencing reads spanning a certain position (coverage), misincorporation rate of non-complementary dNTPs (mismatch rate) and primer extension arrest in reverse transcription (arrest rate). Therefore, a specific pattern composed of arrest and mismatch rate, so-called RT signature, was derived from these tables, being individual for every type of modified nucleoside. In contrast, non-modified positions are not expected to show a prominent RT signature, neither in terms of mismatch, nor arrest rate. Along with 17 other descriptive parameters (see Section 2.2), these profile files set the base for further analysis and were used to investigate individual polymerase behavior.

### 3.1. Manganese Ions Increase Error Rate at Non-Modified Sites

To determine the general influence of manganese treatment, we analyzed a dataset comprising previously created profile files of all non-modified sites. Therefore, we chose a boxplot as accumulated representation of all data points, thereby simplifying visualization of both, median and average values for the respective features in reference (0 mM = 3 mM MgCl_2_) and treated (0.5 to 5 mM MnCl_2_) samples. Additionally, a violin plot, differing in width, depending on data density at this point, was used to display the jump rate, as a boxplot was not applicable.

**Figure 2:**
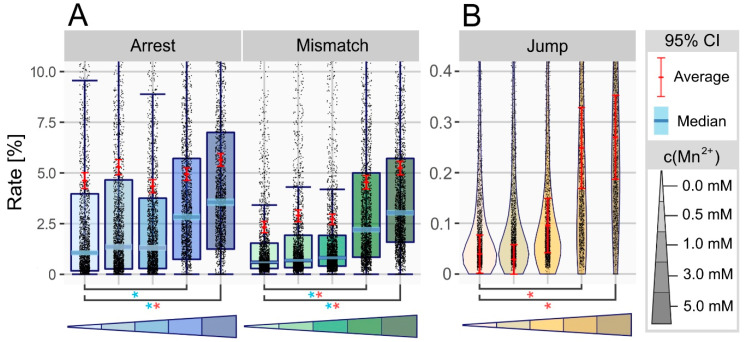
(**A**) Boxplot showing the RT signature of all non-modified sites in tRNA of *S. cerevisiae* at differing manganese concentrations, using EpiScript RT. The triangled color palette represents increasing concentrations of Mn^2+^, with black dots accounting for single data points. The 95% confidence intervals are displayed in light blue (median) and red (average), with colored asterisk (*) indicating significant differences (*p*-value < 0.05). (**B**) Violin plot based on the same dataset and color code as (**A**), displaying the jump rate. The width of each plot differs by amount of data at the given axis intercept.

Using data from EpiScript RT as an example, Figure 2A shows a clear increase in all three main features when comparing reference and samples treated with high manganese concentrations. Regarding mismatch and arrest, the increase in median indicated changes on many positions, although the relatively low difference on average points towards small alteration of each position. Going into detail, more than 95% of changes occurred in the low range of 0.5 to 2.5%. Looking at Figure 2B, a clear upward shift of values close to the x-axis is visible as a complete shape-shift of the plot, thereby expressing a general increase in jump rate. Furthermore, the percentage of positions bearing a jump rose from 8% (0 mM) to 13% (3 mM). Consequently, all three parameters displayed a continuous increase towards higher manganese concentrations, with the recorded maximum at 5 mM MnCl_2_. All effects visible for EpiScript RT were in general accordance with results for SuperScript III, SuperScript IV and ProtoScript II, though variant to a smaller extent.

### 3.2. Manganese Facilitates Overcoming of Blockades in Watson-Crick Base Pairing

Taking the overall changes from non-modified sites into account, we investigated how positions bearing a modified nucleoside would react to altered RT conditions. Therefore, a previously created compilation of modified positions based on the Modomics database [5], was analyzed within the newly obtained dataset.

**Figure 3:**
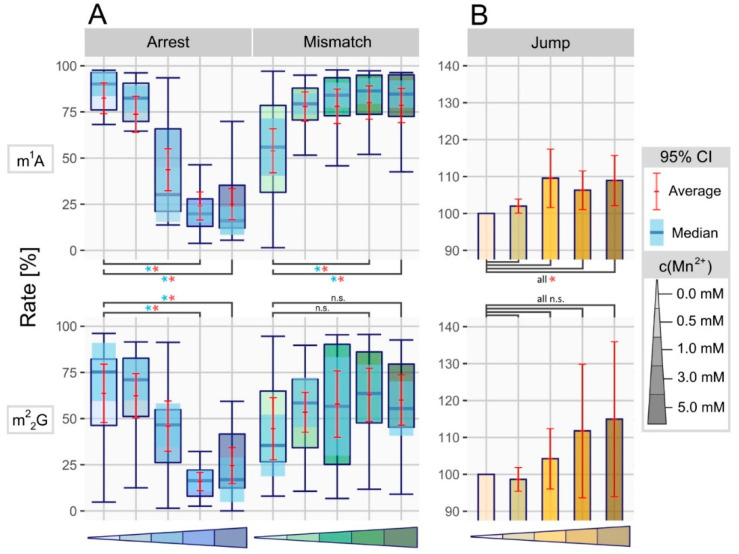
(**A**) Boxplot showing the RT signatures of all m^1^A and m^2^_2_G positions in tRNA from Saccharomyces cerevisiae at differing manganese concentrations, using EpiScript RT. The triangled color palette represents increasing concentrations of Mn^2+^. The 95% confidence intervals are displayed in light blue (median) and red (average), with colored asterisk (*) indicating significant differences (*p*-value < 0.05). (**B**) Normalized bar plot based on the same dataset and color code as (**A**), displaying the difference in jump rate between normalized reference and manganese treated samples.

Normally, RNA modifications such as m^1^A display a strong RT signature, comprising high arrest and mismatch rates of above 50% on most positions (see Table 1). Therefore, with the feature increase for non-modified sites in mind, a further and stronger raise was expected. In contradiction to this, Figure 3A shows a massive decrease in arrest rate for both, m^1^A and m^2^_2_G when comparing reference (3 mM MgCl_2_) and treated (3 mM MnCl_2_) samples, using EpiScript RT for reverse transcription. The average arrest rate dropped from 82% down to 24% for m^1^A, strongly reducing the amount of cDNA that ends at m^1^A positions. As expected, the mismatch rate increased for both modifications, in case of m^1^A from 54% up to 80% average, with misincorporation peaking at m^1^A position 58 in tRNA Leu^TAA^ by rising 53% in total. However, this effect was less pronounced for m^2^_2_G than for m^1^A. Looking at both arrest and mismatch rate, the peak difference is visible at 3 mM MnCl_2_, falling off slightly for further increased concentrations (5 mM). Additionally, similar effects were visible for m^1^G and m^3^C, two frequent RNA modifications, displaying a vast decrease of arrest rate and strongly increased mismatch rates (Figure S3). Going into further detail, the mismatch composition on m^1^A, m^2^_2_G, m^1^G and m^3^C sites was analyzed (Figure S4), as it was previously shown to be heavily dependent on the modification-preceding base of m^1^A sites [22]. We observed a pronounced influence of Mn^2+^-concentration on type and frequency of misincorporated bases, adding to the proposed prebase effect.

### 3.3. Manganese Enhances the Nucleotide Skipping Ability of Reverse Transcriptases

The ability of polymerases to skip certain nucleotides in primer elongation is a rather uncharacterized appearance, which has only recently been described in a RNA modification context [16]. Here, we used the percentage of deletions occurring at, or shortly after the respective position, as another characterizing feature in RT signature, named jump rate.

In contrast to other features, jumps were rare events, occurring on less than 0.6% of non-modified and nearly 19% of modification-associated sites (Mismatch: 16%/31%). Additionally, ribonucleotides with a modified Watson-Crick face induced far more nucleotide skipping, hence displaying jump on 65% of m^1^A positions (Mismatch: 91%). Normally, jump rate is limited to values below 5% with single positions rising to around 20%. However, the Mn^2+^-treated samples showed rates in the range of 50–70% for certain m^1^A positions, leading to an overall significant increase of this feature. The difference in jump rate was calculated for every m^1^A and m^2^_2_G position in reference (0 mM = 3 mM Mg^2+^), compared to the treated samples and is displayed in Figure 3B. We observed significant differences for m^1^A in all treated samples compared to the normalized reference, while m^2^_2_G showed no significant influence of Mn^2+^ treatment. The strongest alteration of a single position was visible for m^1^A at position 58 in tRNA Asn^GTT^ of the 1 mM treated specimen. A raise in jump rate from 4% up to 49% was observed, thus drastically reducing the arrest rate in cDNA synthesis.

### 3.4. Manganese-Derived Alteration of RT Performance Is Polymerase-Specific

**Figure 4:**
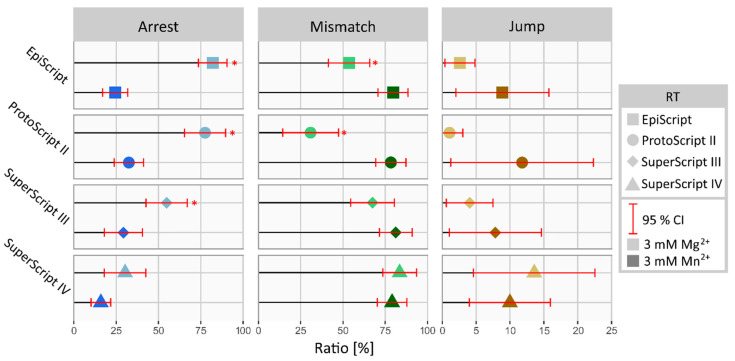
Comparative plot of all used polymerases breaking down their average profile for m^1^A positions in *S. cerevisiae* tRNA. The 95% confidence interval is displayed in red, with colored asterisk (*) indicating significant differences (*p*-value < 0.05) between Mg^2+^ and Mn^2+^ conditions. Enzymes are displayed in different shapes with colors signaling Mg^2+^ (pale) or Mn^2+^ (intense) treatment.

Every polymerase displays an individual behavior in reverse transcription when processing modified and non-modified positions. This is visible in RT signature, where different compositions of arrest, mismatch and jump rate occur, dependent on both, the enzyme and processed nucleoside. We investigated if manganese induced alteration differed between transcriptases by comparing their performance on *S. cerevisiae* tRNA under equimolar concentrations of Mg^2+^ and Mn^2+^. As shown in Figure 4 for a set of all m^1^A positions, manganese treated samples in general showed a lower arrest rate while ranging at higher mismatch levels. Looking at the jump rate, no significant increase could be observed, even though an overall tendency towards enhanced nucleotide skipping was visible. EpiScript and ProtoScript II RT showed significant differences in both arrest and mismatch rate, with the first also applying to SuperScript III. The SuperScript IV in contrast showed already high levels for reference conditions, with a fairly low arrest rate when compared to other polymerases. Unlike the other RTs, SuperScript IV displayed slightly decreased mismatch and jump rate under manganese treatment, hence increasing sequence fidelity.

## 4. Discussion and Conclusions

In this work, we examined the influence of manganese ions on reverse transcription and therefrom derived RNA modification analysis by performing reverse transcription with four different RTs, thereby replacing MgCl_2_ with various concentrations of MnCl_2_. The presented data reveal a profound influence of manganese ions on reduced sequence fidelity and an altered rate of primer extension arrest in cDNA synthesis. Furthermore, the number of deletions visible in sequencing data increased drastically, thus implying an enhanced read-through capability as effect of Mn^2+^ on the reverse transcriptase by promoting nucleotide skipping over synthesis abortion. These effects were less pronounced for non-modified nucleosides, while distinct to modifications such as m^1^A or m^2^_2_G that bear a modified Watson-Crick face, thus impeding conventional base pairing. While known to inflict primer extension arrest during reverse transcription, these modifications showed significantly reduced arrest rates under manganese influence and increased misincorporation and deletion levels instead. However, these effects were individual to both, the modification type and reverse transcriptase, hence not observable for every modification and polymerase. Nevertheless, the increased read-through rate could be beneficial in experiments restricted to library preparations that are incapable of capturing abortive cDNA fragments. Additionally, misincorporation and deletion rates have been previously identified as key factors in automated modification calling based on random forest models, displaying higher performance on datasets with increased read-through [16]. Considering all these findings we strongly suggest that a MnCl_2_-based reverse transcription could be beneficial for RT signature analysis, thereby increasing the read-through rate and thus obtaining stronger signals. This approach and the associated increased signal-to-noise ratio is currently applied in m^1^A quantification of mitochondrial RNA species (unpublished results). Furthermore, the rising number of deletions and therefrom altered jump rate could serve as major feature for machine learning-based methods in modification calling. Together with prudent analysis of modification-specific differences between conventional and manganese-altered transcription patterns, this combination may help further developing RNA modification analysis.

## Figures and Tables

**Table 1:** Exemplary profile file of 10 m^1^A positions from the EpiScript reference sample after final data treatment.

Ref_Seg	Mod	Pos	Ref_Base	Cov	Pre_Base	Mism_Rate	A	G	T	C	N	a	g	t	c	n	Single_Jump_Rate_Direct	Single_Jump Rate_Delayed	Double_Jump_Rate	Arrest_Rate
tdbR00000369|Saccharomyces_cerevisiae|4932|Arg|ACG	m1A	58	A	167	G	0.94012	10	39	24	7	0	0	58	25	4	0	0.00000	0.01754	0.01754	0.23661
tdbR00000370|Saccharomyces_cerevisiae|4932|Arg|TCT	m1A	57	A	25	C	0.52000	12	0	0	6	1	0	0	0	6	0	0.00000	0.00000	0.00000	0.03846
tdbR00000300|Saccharomyces_cerevisiae|4932|Asn|GTT	m1A	59	A	553	C	0.93671	35	4	7	229	1	0	5	11	261	0	0.00318	0.08493	0.40552	0.09519
tdbR00000021|Saccharomyces_cerevisiae|4932|Cys|GCA	m1A	57	A	562	T	0.94128	33	12	205	17	2	0	18	266	9	0	0.00000	0.00000	0.00858	0.09048
tdbR00000170|Saccharomyces_cerevisiae|4932|Ile|AAT	m1A	59	A	1177	T	0.96517	41	15	447	74	4	0	16	540	40	0	0.00168	0.00168	0.00587	0.23181
tdbM00000006|Saccharomyces_cerevisiae|4932|Ile|TAT	m1A	58	A	447	T	0.85682	64	15	138	23	1	0	25	161	20	0	0.00000	0.00665	0.00000	0.46437
tdbR00000251|Saccharomyces_cerevisiae|4932|Leu|TAA	m1A	69	A	571	A	0.35902	366	16	58	57	1	0	13	37	23	0	0.00520	0.00000	0.00173	0.43098
tdbR00000250|Saccharomyces_cerevisiae|4932|Leu|TAG	m1A	67	A	544	A	0.55699	241	17	96	41	2	0	22	87	38	0	0.01961	0.00000	0.00713	0.26988
tdbR00000192|Saccharomyces_cerevisiae|4932|Lys|CTT	m1A	58	A	604	G	0.72682	165	90	85	12	2	0	127	114	9	0	0.03343	0.00304	0.04407	0.17955
tdbR00000193|Saccharomyces_cerevisiae|4932|Lys|TTT	m1A	58	A	257	G	0.86381	35	36	53	9	1	0	46	69	8	0	0.07190	0.00000	0.06863	0.19895

Features include coverage (cov), arrest (arres_rate) and jump rates (single_jump_rate_direct, single_jump_rate_delayed, double_jump_rate). Additionally, reference name (ref_seg), reference position (pos), reference base (ref_base) and coverage at the respective position were shown in the profile. Complementary, detailed information on the number of aligned bases by type (A, C, G, T) and unknown read bases (N), differentiated for forward (capital letters—A, C, G, T, N) and reverse reads (small letters—a, c, g, t, n), as well as the 3’-preceding base (prebase) were included.

## Supplementary Materials

The following are available online at https://www.mdpi.com/2073-4425/11/8/950/s1. Figure S1: Graphical overview of modification profile extraction. Figure S2: Exemplary alteration of m^1^A signatures. Figure S3: RT signature alteration of m^1^G and m^3^C. Figure S4: Manganese influence of mismatch composition on modification sites.
