# Supplementary material for: Manganese Ions Individually Alter the Reverse Transcription Signature of Modified Ribonucleosides
Source: Genes (Basel). 2020 Aug 18;11(8):950. doi: 10.3390/genes11080950 (PMC7466121; doi:10.3390/genes11080950)
Supplement: Supplementary file 1 [file genes-11-00950-s001.zip › Supplement/Supplementary Materials.docx]

Supplementary Materials:


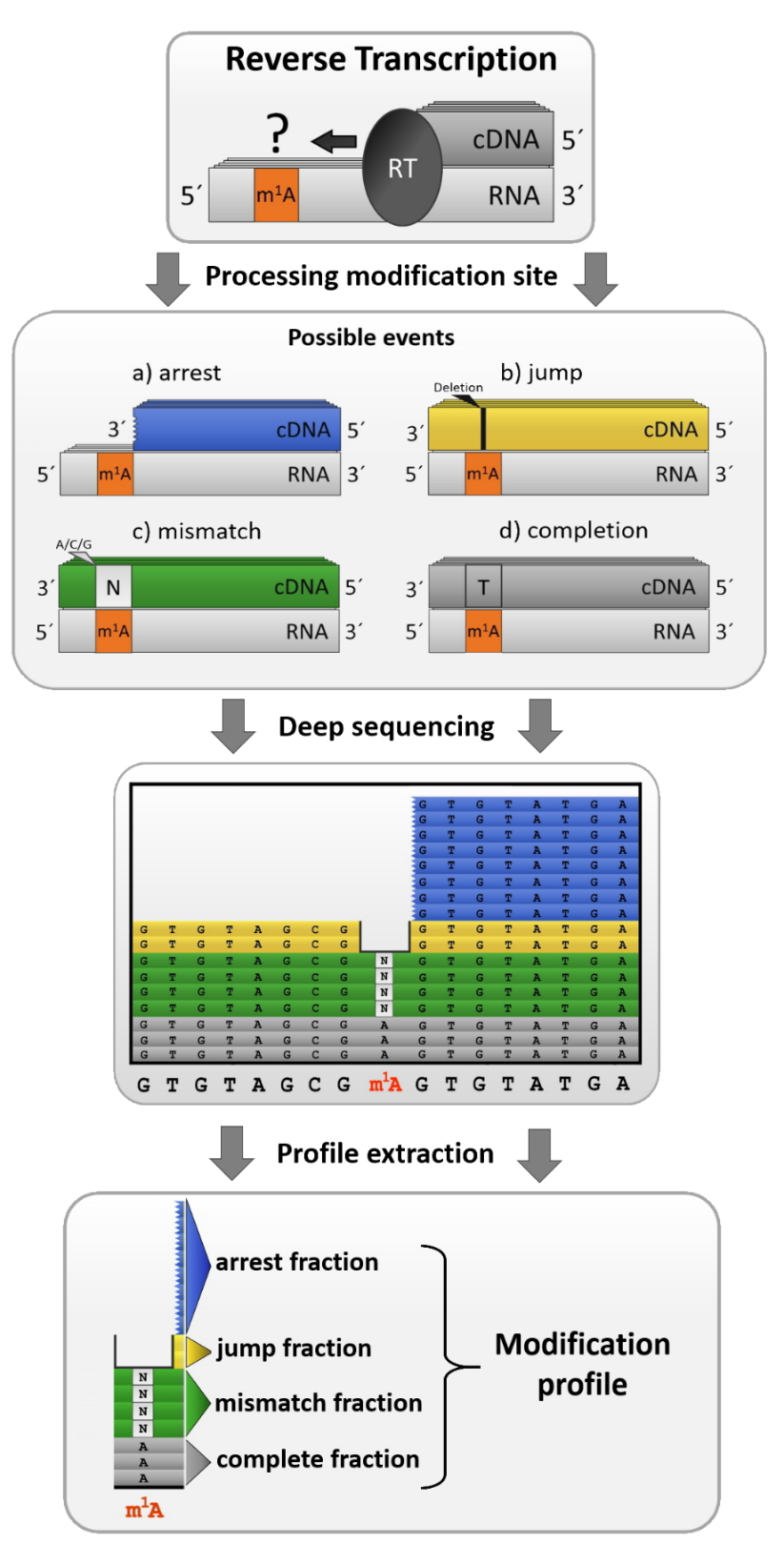
**Figure S1.** Graphical overview of RT signature alteration and modification profile extraction from RNA-seq data at an exemplary m^1^A site. During reverse transcription, the cDNA can either be completed as a full-length complementary strand, or bear one of three possible alterations (a-d), inflicted by the modified Watson-Crick face of m^1^A. Following library preparation, retrieved sequencing output is processed in an automated pipeline, yielding a profile file as presented in Table 1. The coverage is defined as the total number of reads (jump, mismatch and complete) covering the respective position. In contrast, the arrest rate is calculated as the quotient of arrest fraction and coverage of the previous (N+1) position. Furthermore, the jump rate is calculated in relation to the total coverage of the respective site, while the mismatch rate is defined as the quotient of mismatch fraction and remnant coverage (mismatch and complete). As a result, the sum of jump and mismatch rate can exceed 100%, as visible in Figure S2**.**


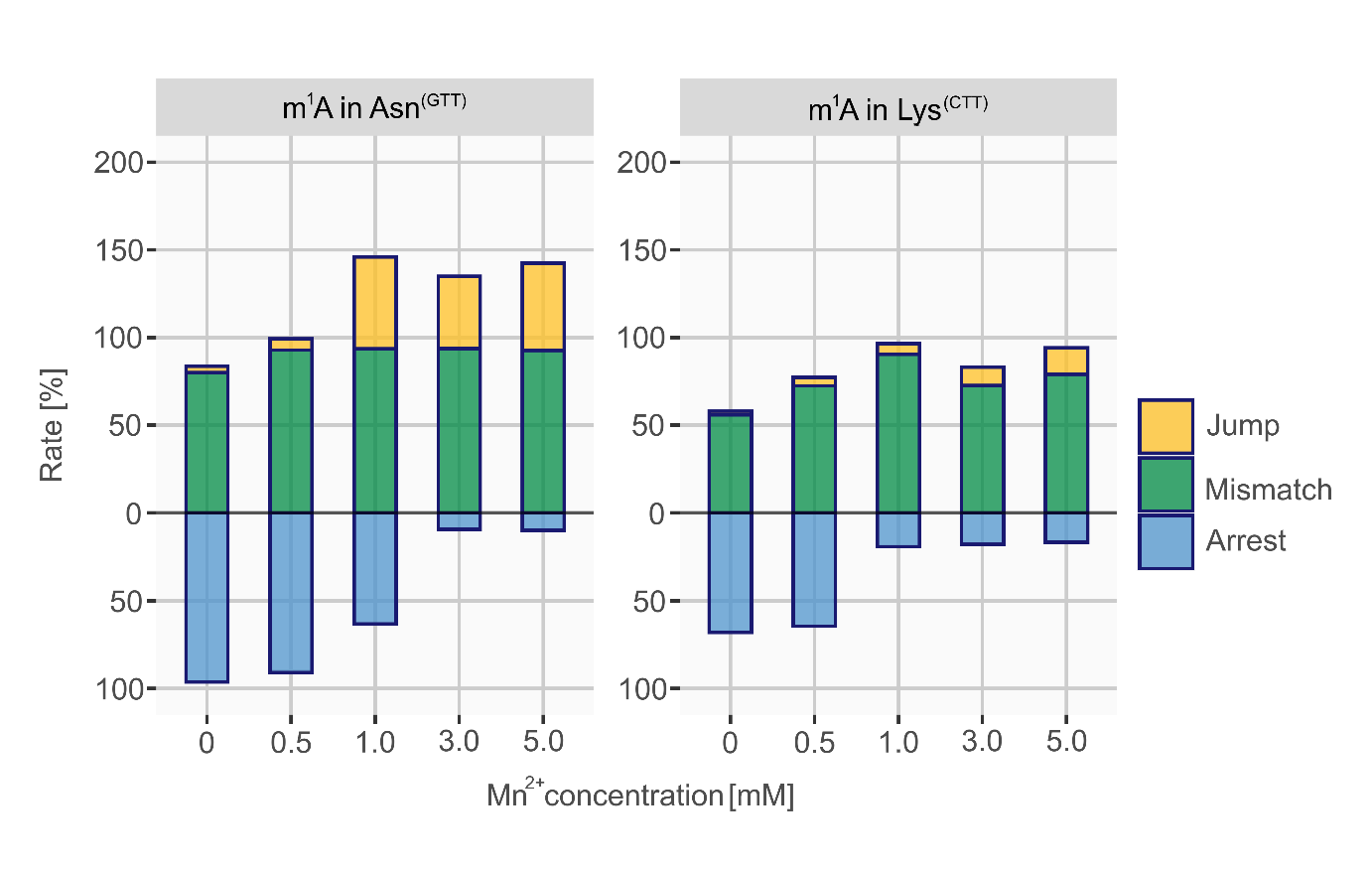
**Figure S2.** Comparison of two sets of RT signatures from m^1^A positions in tRNA Asn^(GTT)^ and Lys^(CTT)^, processed with EpiScript RT under reference (3 mM Mg^2+^) and altered (0.5, 1.0, 3.0, 5.0 mM Mn^2+^) conditions. As jump, mismatch and arrest rate are calculated differently (see Figure S2), rates over 100% in total are visible.


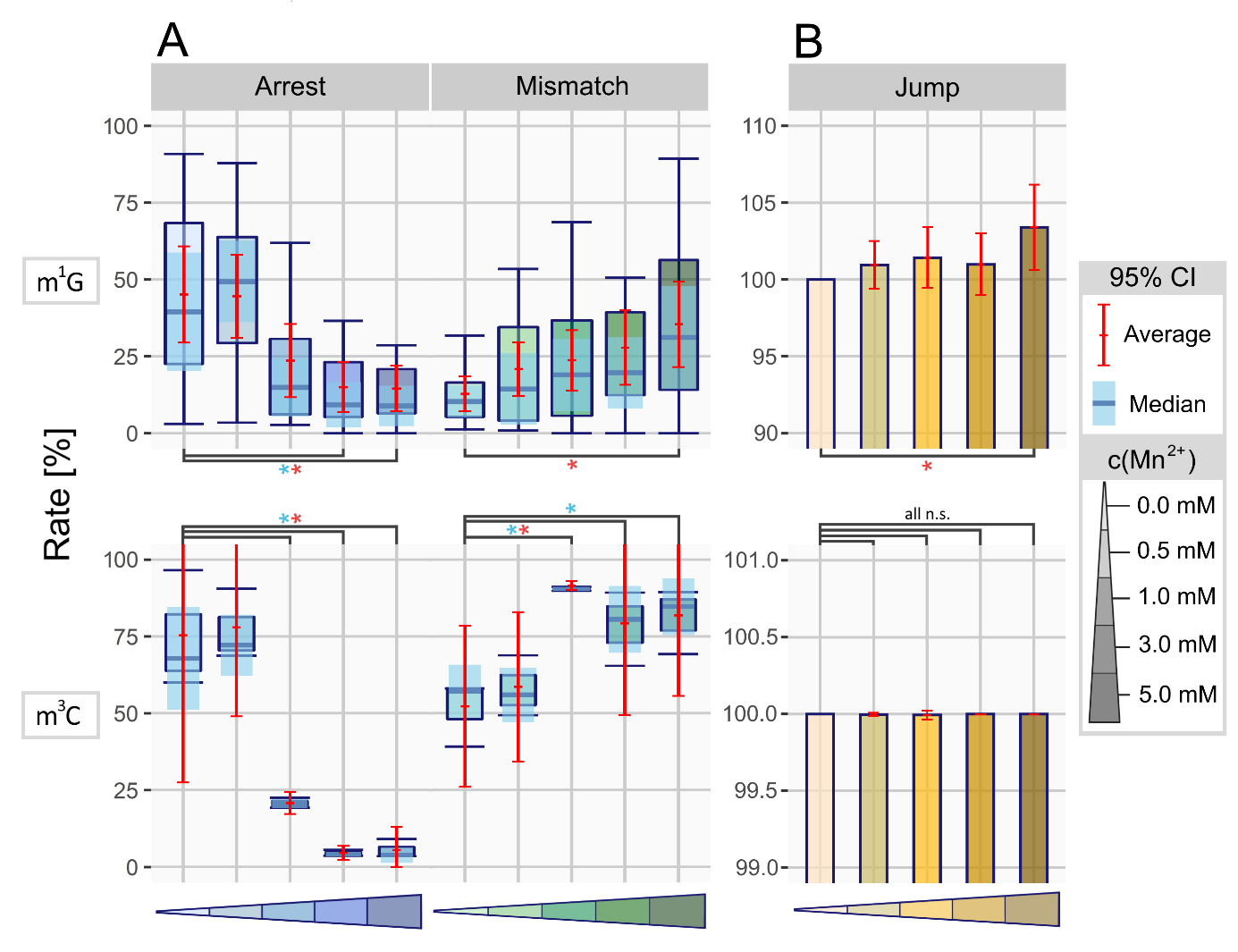
**Figure S3.** **(A)** Boxplot showing the RT signatures of all m^1^G and m^3^C positions in tRNA from Saccharomyces cerevisiae at differing manganese concentrations, using EpiScript RT. The triangled colour palette represents increasing concentrations of Mn^2+^. The 95% confidence intervals are displayed in light blue (median) and red (average), with colored stars indicating significant differences. **(B)** Normalized bar plot based on the same dataset and color code as (A), displaying the difference in jump rate between normalized reference and manganese treated samples.


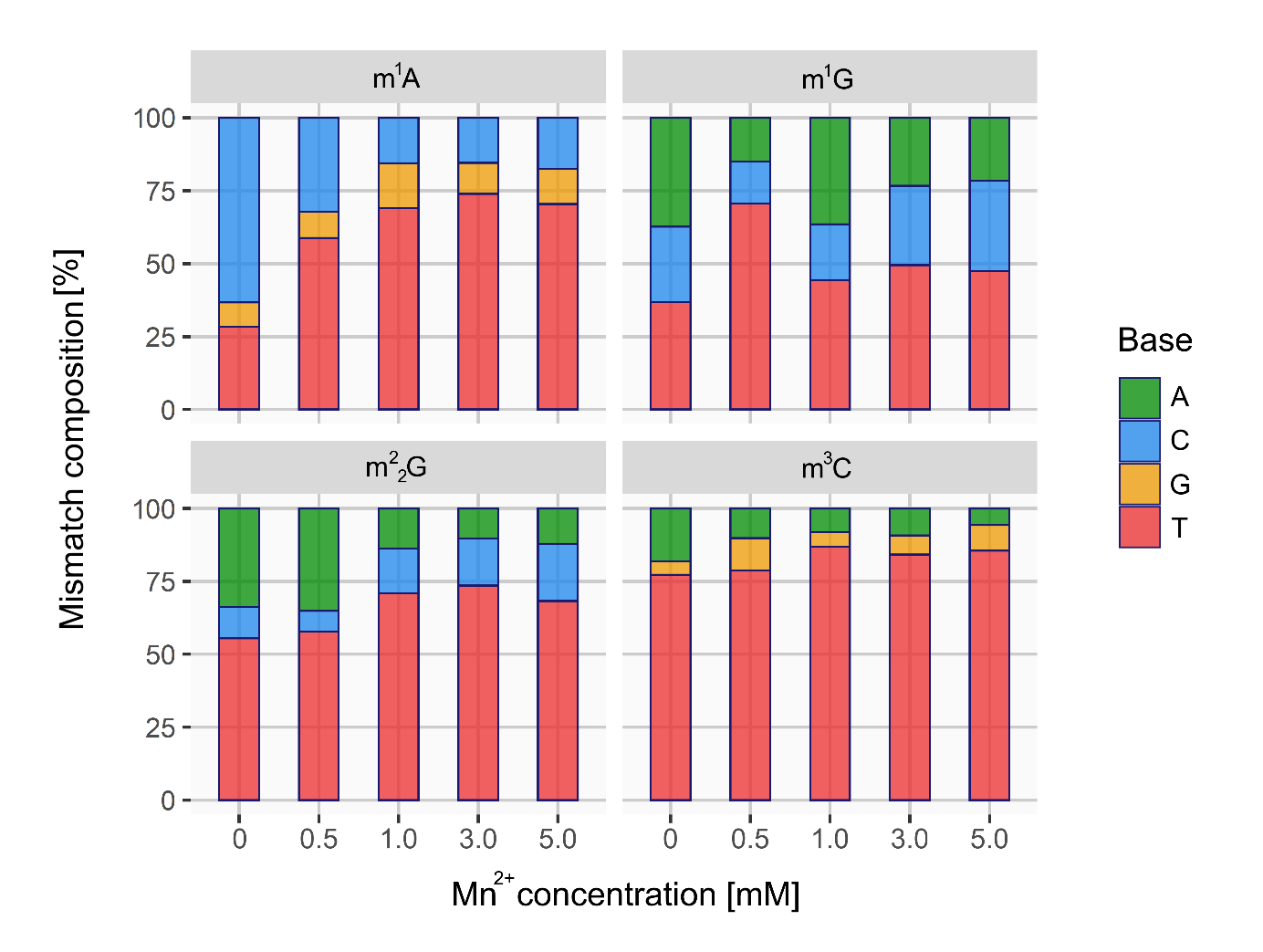
**Figure S4.** Overview of normalized mismatch composition on modification sites from S.cerevisiae tRNA, processed with EpiScript RT, displaying impact of altered transcription conditions on type and frequency of misincorporated bases.
