# Supplementary figures and images for: Manganese Ions Individually Alter the Reverse Transcription Signature of Modified Ribonucleosides
Source: Genes (Basel). 2020 Aug 18;11(8):950. doi: 10.3390/genes11080950 (PMC7466121; doi:10.3390/genes11080950)

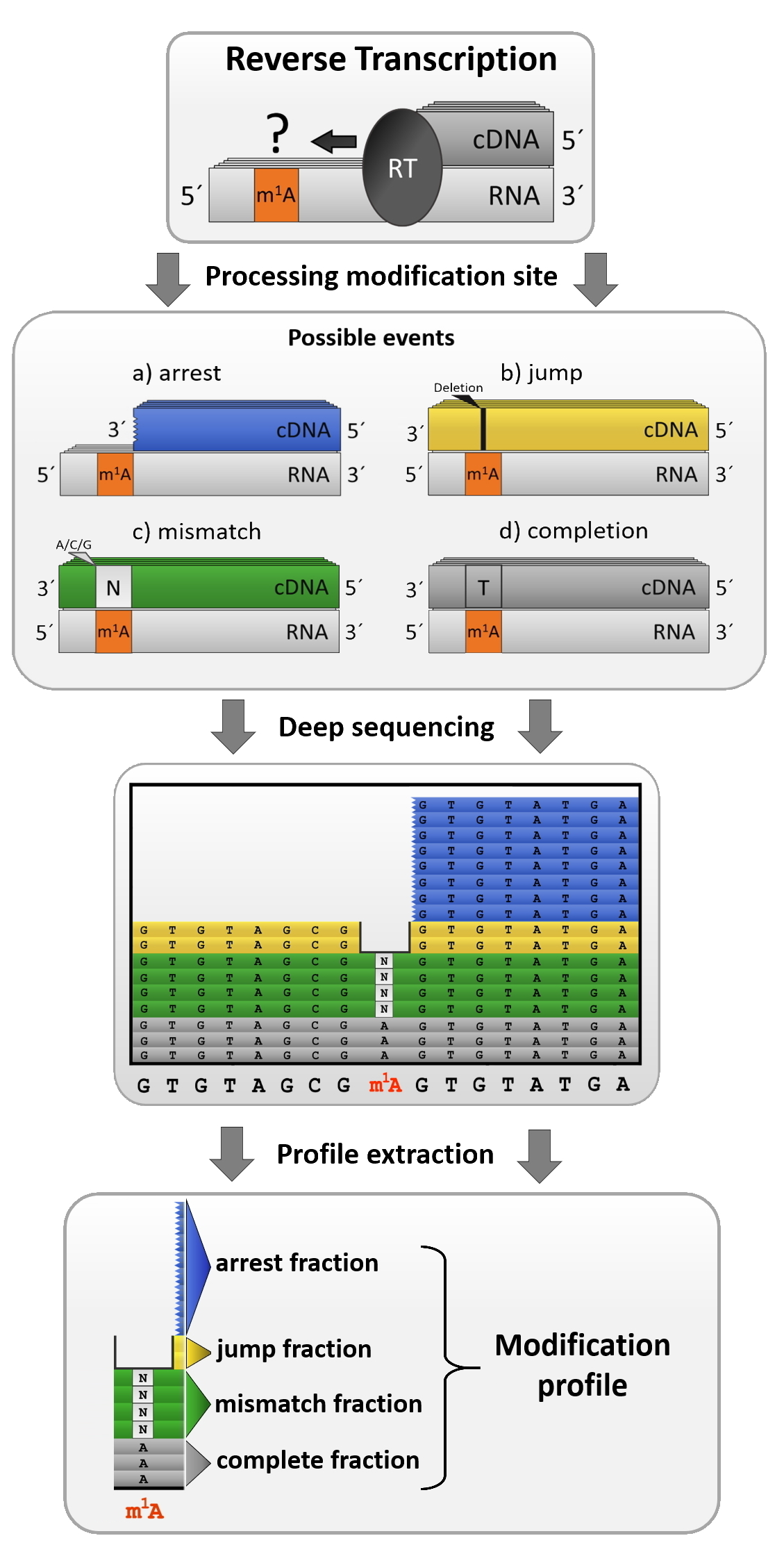

Supplement: Supplementary file 1 [file genes-11-00950-s001.zip › Supplement/Figure_S1.png]

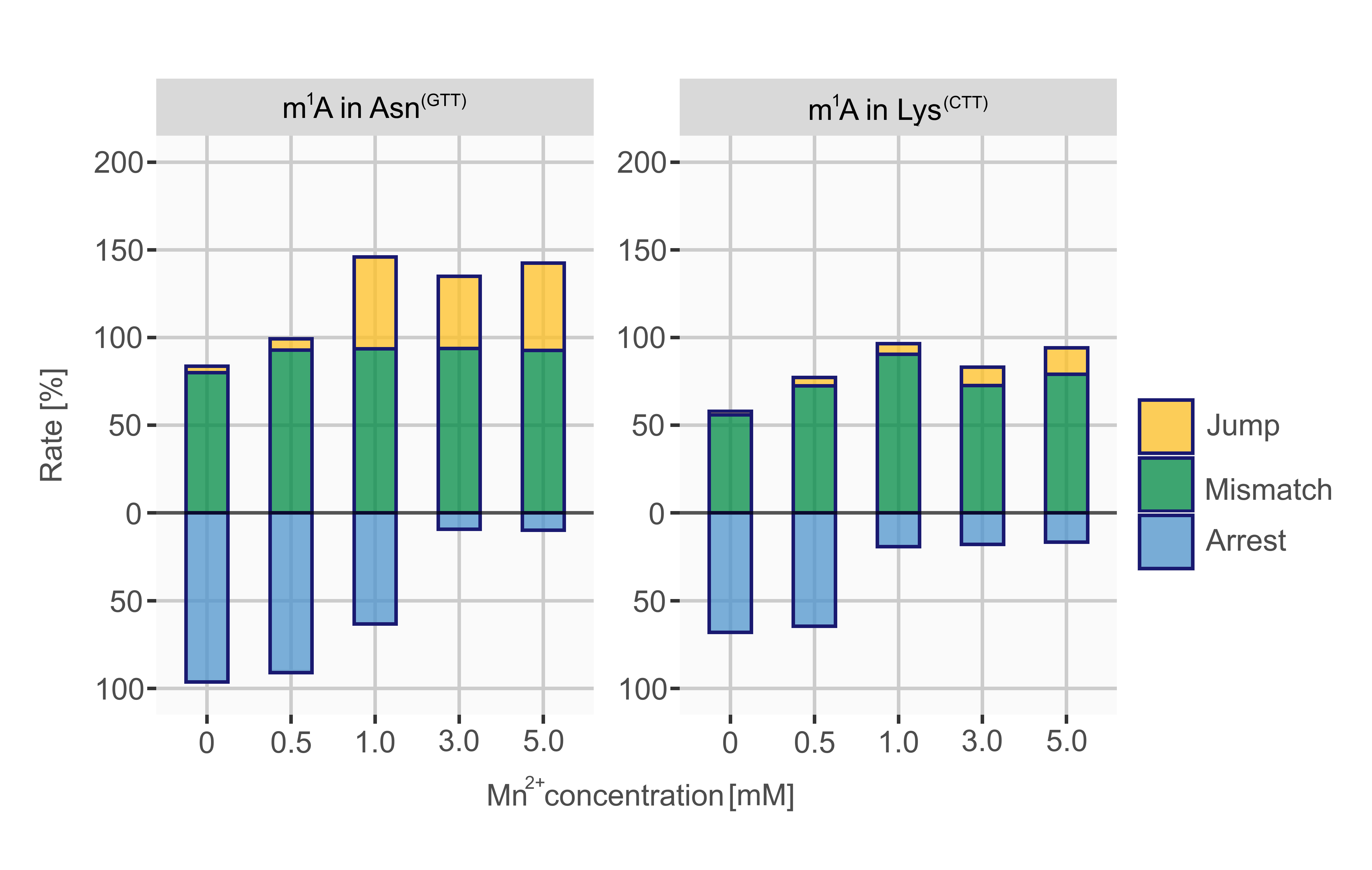

Supplement: Supplementary file 1 [file genes-11-00950-s001.zip › Supplement/Figure_S2.png]

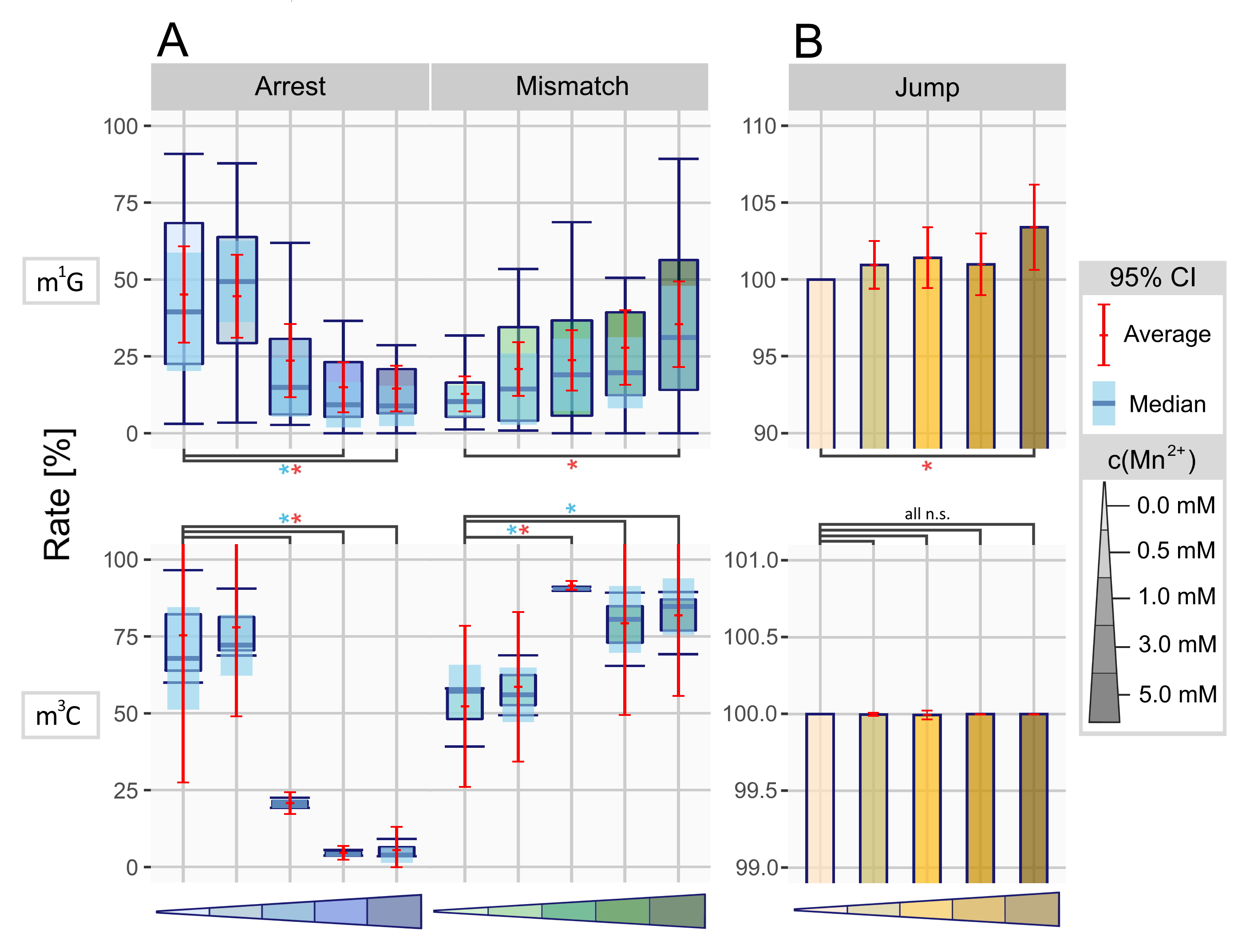

Supplement: Supplementary file 1 [file genes-11-00950-s001.zip › Supplement/Figure_S3.png]

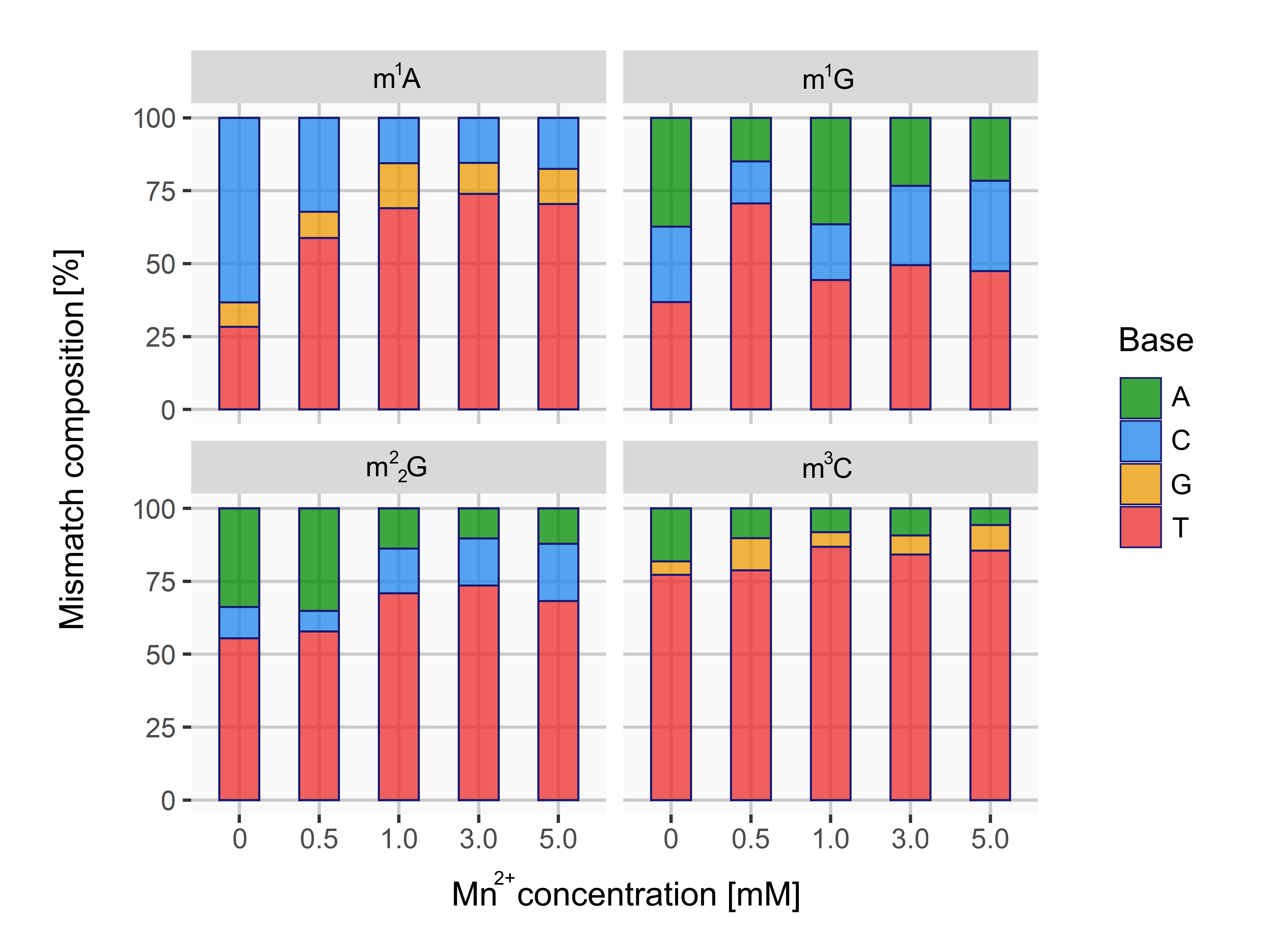

Supplement: Supplementary file 1 [file genes-11-00950-s001.zip › Supplement/Figure_S4.png]
